# Practical Validation of United States Centers for Disease Control and Prevention Assays for the Detection of Human Respiratory Syncytial Virus in Pediatric Inpatients in Japan

**DOI:** 10.3390/pathogens11070754

**Published:** 2022-07-01

**Authors:** Reiko Suwa, Yohei Kume, Miyuki Kawase, Mina Chishiki, Takashi Ono, Sakurako Norito, Ko Sato, Michiko Okamoto, Satoru Kumaki, Yukio Nagai, Mitsuaki Hosoya, Makoto Takeda, Hidekazu Nishimura, Koichi Hashimoto, Kazuya Shirato

**Affiliations:** 1 Department of Virology III, National Institute of Infectious Diseases, 4-7-1 Gakuen, Musashimurayama 208-0011, Tokyo, Japan; r-suwa@niid.go.jp (R.S.); kawase@niid.go.jp (M.K.); mtakeda@niid.go.jp (M.T.); 2 Department of Pediatrics, School of Medicine, Fukushima Medical University, 1 Hikariga-Oka, Fukushima 960-1295, Fukushima, Japan; kumetti@fmu.ac.jp (Y.K.); chishiki@fmu.ac.jp (M.C.); takashi5@fmu.ac.jp (T.O.); snorito@fmu.ac.jp (S.N.); mhosoya@fmu.ac.jp (M.H.); don@fmu.ac.jp (K.H.); 3 Virus Research Center, Clinical Research Division, Sendai Medical Center, National Hospital Organization, 2-11-12 Miyagino, Miyagino-ku, Sendai 983-8520, Miyagi, Japan; ko-sato@med.tohoku.ac.jp (K.S.); okamotom@med.tohoku.ac.jp (M.O.); hide_nishimura1955@yahoo.co.jp (H.N.); 4 Department of Pediatrics, Sendai Medical Center, National Hospital Organization, 2-11-12 Miyagino, Miyagino-ku, Sendai 980-8575, Miyagi, Japan; kumaki.satoru.zh@mail.hosp.go.jp; 5 Nagai Pediatric Clinic, 1-25-10 Miyagino, Miyagino-ku, Sendai 983-0045, Miyagi, Japan; nagai-children-c@circus.ocn.ne.jp

**Keywords:** human respiratory syncytial virus (RSV), human orthopneumovirus, real-time RT-PCR, severe acute respiratory infection, global surveillance

## Abstract

The World Health Organization initiated a global surveillance system for respiratory syncytial virus (RSV) in 2015, and the pilot surveillance is ongoing. The real-time RT-PCR RSV assays (Pan-RSV and duplex assays) developed by the United States Centers for Disease Control and Prevention are applied as the standard assays. To introduce these as standard assays in Japan, their practicality was evaluated using 2261 specimens obtained from pediatric inpatients in Japan, which were collected from 2018 to 2021. Although the Pan-RSV and duplex assays had similar analytical sensitivities, they yielded 630 (27.9%) and 786 (34.8%) RSV-positive specimens, respectively (*p* < 0.001). Although sequencing analysis showed mismatches in the reverse primer used in the Pan-RSV assay, these mismatches did not affect its analytical sensitivity. The analysis of read numbers of RSV isolates from air–liquid interface culture of human bronchial/tracheal epithelial cells showed that the duplex assay had a greater number of reads than did the Pan-RSV assay. Therefore, the duplex assay has superior detection performance compared with the Pan-RSV assay, but the two assays have similar analytical sensitivities.

## 1. Introduction

Human orthopneumovirus, also known as human respiratory syncytial virus (RSV), causes respiratory tract infections in children worldwide. Preterm infants and infants with underlying cardiopulmonary disorders have particularly high risks of severe or fatal RSV respiratory tract infections [1,2,3]. In 2015, in anticipation of the development of an effective vaccine for RSV, the World Health Organization (WHO) initiated discussions of a global RSV surveillance system based on the global influenza surveillance and response system (GISRS) [4]. Pilot surveillance programs were launched in 2016 and 2019; these programs targeted individuals of all ages with influenza-like illness (ILI), acute respiratory infection, or severe acute respiratory infection (SARI) without fever [5,6]. A real-time RT-PCR (Pan-RSV) assay, developed by the United States Centers for Disease Control and Prevention (US-CDC) [7], was used as the standard in the first pilot surveillance program. The Pan-RSV assay can simultaneously detect RSV subgroups A and B, but it requires sequence analyses for subgrouping of positive specimens [7]. Recently, the US-CDC developed a duplex assay for the simultaneous detection of both subgroups of RSV [8]. Although sequencing is not required for subgrouping, two fluorescence probes are required.

There is no standard protocol for real-time RT-PCR assays in the national surveillance of RSV in Japan. With the start of global surveillance as a turning point, preparation of a standard assay for RSV detection has been discussed in Japan. For in-house assays, local validation is important because the available reagents, instruments, and primer/probe qualities can differ among countries. Here, at the national laboratory in Japan, we evaluated two US-CDC assays using specimens from inpatients with respiratory infections for possible adoption as standard assays.

## 2. Results

### 2.1. Assay Conditions 

In Japan, the detection of influenza virus by real-time RT-PCR is performed in accordance with the WHO’s protocol [9]. Therefore, during the surveillance of SARI and ILI, the PCR amplification conditions should follow the WHO’s protocol to enable simultaneous detection of multiple viruses in one plate. In this study, the amplification conditions of the AgPath-ID reagent in the Flu WHO protocol were used in US-CDC assays for RSV detection. The performance of the Pan-RSV assay using the AgPath-ID reagent was evaluated in a previous study; a Cp value of 35 was used as the cutoff value in that study to exclude false-positives caused by nonspecific amplification [10]. It was confirmed that the Pan-RSV assay with AgPath-ID reagent could detect 10 or fewer copies of control RNA in the reported amplification conditions [8] with the same cutoff value (8.34 ± 2.71 copies for A and 6.83 ± 3.95 copies for B, *n* = 6). To evaluate the specificity of the duplex assay, we used clinical specimens that comprised 37 with no virus and 49 with a single virus (37 severe acute respiratory syndrome coronavirus 2, two adenovirus, two human metapneumovirus, two influenza A virus, two human respirovirus 1, two human rubulavirus 2, and two human respirovirus 3). In 86 clinical specimens, amplification signals (two for A and six for B) were shown, and the average Cp values were 38.16 and 38.7, respectively. Amplification signals were also seen in negative controls (one for A and two for B), and the minimum Cp value was 37.09 (*n* = 8 for each probe). Therefore, a Cp value of 37 was used as the cutoff to distinguish false-positives, although a Cp value of 36.5 was required to exclude false-positives in the original amplification condition [8], with analytical sensitivities of 4.82 ± 3.58 copies for A and 15.3 ± 11.3 copies for B (*n* = 4). In the WHO protocol, the analytical sensitivities of the Pan-RSV assay were 1.86 ± 2.78 copies for subgroup A and 4.64 ± 4.54 copies for subgroup B; the analytical sensitivities of the duplex assay were 6.18 ± 3.18 copies for subgroup A and 7.04 ± 2.75 copies for subgroup B (*n* = 4). There were no significant differences between subgroups (Pan-RSV, *p* = 0.335; duplex, *p* = 0.638) or between the Pan-RSV and duplex assays (subgroup A, *p* = 0.088; subgroup B, *p* = 0.076). These were not inferior to the original results [8], suggesting that altered amplification conditions did not affect the quality of the assays.

### 2.2. Evaluation of the US-CDC Assays 

The Pan-RSV and duplex assays were used to detect RSV in clinical specimens that had been collected from pediatric inpatients in Fukushima, Japan, from 2018 to 2021. In total, 2261 nasopharyngeal swabs were tested; 630 were positive according to the Pan-RSV assay (27.9%) and 786 were positive according to the duplex assay (34.8%) (*p* < 0.001). Of the 786 with positive duplex assay results, 416 (52.9%) were subgroup A and 370 (47.1%) were subgroup B (*p =* 0.023). Among the 630 with positive Pan-RSV assay results, 341 (58.0%) subgroup A and 248 subgroup B (42.2%) were detected by the duplex assay (*p* < 0.001). The detection rate of the duplex assay was higher than the Pan-RSV assay for both subgroups (subgroup A, *p* = 0.003; subgroup B, *p* < 0.001).

### 2.3. Sequencing of the Pan-RSV Assay Target Region 

Nucleotide mismatches in primer/probe sequences may affect analytical sensitivity; therefore, the mismatches were analyzed using about 3000 nearly complete genomic sequences from GenBank (containing both subgroups A and B) (Table 1). About 98% of the sequences did not have mismatches for the forward primer of the Pan-RSV assay. In contrast, about half of the sequences had mismatches for the reverse primer and the probe of the Pan-RSV assay. A mismatch within 10 nucleotides from the 3′-end of the primer could affect the amplification efficiency. Most of the mismatches in the reverse primer were G to A at nucleotide 12 from the 5′-end (1299, 44.3%). However, A to G at nucleotide 24, which was five nucleotides from its 3′-end, increased after 2016. About 97–98% of the sequences did not have any mismatches for primers in the duplex assay, and the existing mismatches were not close to the 3′-end. This suggested the occurrence of genetic changes in the Pan-RSV-targeted regions.

**Figure 1:**
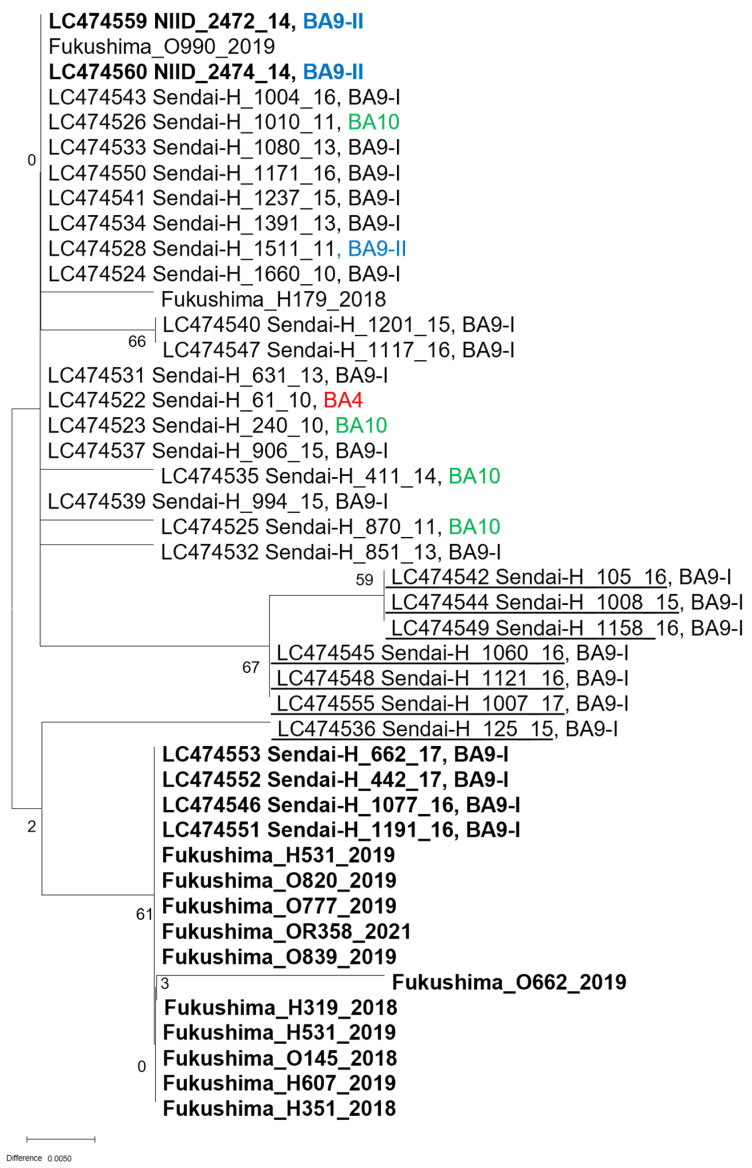
Phylogenetic analysis of the Pan-RSV target sequences using MEGA-X software. The tree was created using the maximum-likelihood method with bootstrap analysis (500 times). Bold, sequence with an A to G substitution at nucleotide 24 of the forward primer used in the Pan-RSV assay. Underlined font, sequences with mismatch(es) to the probe sequences.

To confirm the genetic change in Japan, the Pan-RSV assay target region was sequenced using PCR amplicons from clinical specimens. Ten of the subgroup A specimens (2014–2021) were sequenced. All sequences had two nucleotide mismatches (T to C at nucleotide 17 and C to T at nucleotide 11) with the probe (Table 2). Nearly all subgroup A sequences had no mismatches with the primer sequences, except for RSV/A/NIID/2367/14, which had a mismatch with the forward primer (T to C at nucleotide 9). For subgroup B, 42 of the specimens (2018–2021) were sequenced, and 36 of the almost-complete genome sequences obtained from Sendai between 2010 and 2017 (GenBank accession nos. LC474522–LC474560) [11] were included in the analysis. A mismatch (G to A at nucleotide 12) was present in the forward primer in all analyzed sequences (Table 3). The mismatch in the forward primer (A to G at nucleotide 24) was discovered in 46 of the analyzed sequences, and, similar to the in-silico analysis, it started to appear in 2016 and became dominant in 2018 in Japan. A phylogenetic analysis of the Pan-RSV assay-target sequences yielded three groups, although most sequences were classified into the BA9-I linage based on the G protein sequence (Figure 1). The effect of these mismatches on analytical sensitivity was determined using synthesized RNA obtained from PCR amplification of mismatched sequences (Table 4). None of the mismatches affected the analytical sensitivity of the Pan-RSV assay, although the A to G substitution at nucleotide 24 in the forward primer was five nucleotides from its 3′-end.

### 2.4. Analysis of Read Coverage of the RSV Isolates 

To examine the cause of the lower detection rates with the Pan-RSV assay compared to the duplex assay, the sequence coverage of the targeted region in the RSV genome was analyzed using next-generation sequencing (NGS). Direct NGS analysis of the clinical specimens is ethically prohibited to avoid disclosure of personal information. Therefore, RSV was isolated from the clinical specimens using air–liquid interface (ALI) cultures of human bronchial/tracheal epithelial cells (HBTECs) [12,13], and the cell wash of RSV-infected HBTEC-ALI cultures was used to mimic the clinical swabs. For RSV sequencing, an NGS method based on PCR amplicons has been reported [14]. Although this method avoids direct sequencing of clinical specimens, the whole genomic sequence is amplified by overlapping in six regions and is analyzed using the mixture of amplicons. Therefore, the read numbers do not reflect the original copy numbers. In this study, libraries were directly prepared from the RNA, and read mapping was performed as described in the *Methods* section (Table 5). The target of the Pan-RSV assay is near the 5′-end of the mRNA for M protein, while the target of the duplex assay is near the 5′-end of the mRNA for N protein. The mean read coverages were lower for the Pan-RSV-targeted sequence and the mRNA for M protein than for the duplex assay-targeted sequence and the mRNA for N protein, suggesting that the amount of targeted region sequence contributes to the dissimilar detection rates in the Pan-RSV and duplex assays.

## 3. Discussion

The Pan-RSV assay was used for the first WHO pilot RSV surveillance program, with the ultimate goal of developing a global surveillance system based on the GISRS [4]. The Pan-RSV assay has superior performance and high sensitivity, and it is cost effective if a lot of undiagnosed specimens require testing because it can simultaneously detect RSV subgroups A and B [7]. However, sequence analyses are required for RSV subgrouping [7], claiming another resource for genetic analysis to monitor trends in the subgroups. In the second pilot surveillance program, a duplex assay developed by the Victorian Infectious Diseases Reference Laboratory was used to replace additional genetic analysis for subgrouping. However, the assay requires a SensiFast Probe Lo-ROX One-Step kit (Bioline, London, UK) or TaqMan Fast Virus 1-Step kit (Thermo Fisher Scientific, Waltham, MA, USA) to prepare the real-time RT-PCR master mix [15]. Thus, both US-CDC assays have been validated through two pilot surveillance programs, although Japan did not participate in these. Therefore, they were validated in this study considering ILI surveillance in Japan. The US-CDC duplex assay showed superior RSV detection performance compared with the Pan-RSV assay because of differences in the number of target reads. In addition, the subgroup B sequences targeted by the Pan-RSV assay demonstrated mismatches with the primer/probe sequences; these mismatches do not affect the analytical sensitivity of the assay, but additional mismatches may lead to reduced analytical sensitivity. Therefore, the Japanese RSV surveillance system should be based on the duplex assay.

The present study also showed that genetic variations in the M gene, which is the targeted region in the Pan-RSV assay, emerged in the mid-2010s. In molecular epidemiological studies of RSV, the sequences in the attachment glycoprotein (G protein) and/or fusion (F) protein are typically used for genotyping because they are the main targets for host immunity. In particular, G is preferred because it contains a hypervariable region [16,17,18]. Although most sequences used in this study belonged to BA9-1 based on the G protein sequence, phylogenetic analysis of Pan-RSV assay-targeted sequences divided them into three groups based on M gene mismatches. This indicates that the genetic evolution of other regions was missed by routine genotyping of RSV. Certainly, genotyping based on the G and/or F genes is useful to show the correlation between the evolution of viral genes and human immunity. However, it is important to monitor targeted region sequences over time to maintain the performance of real-time RT-PCR-based assays for RSV.

## 4. Materials and Methods

### 4.1. Clinical Specimens 

In total, 2261 nasopharyngeal swabs were collected from pediatric inpatients aged <15 years with respiratory tract infections, in Fukushima, Japan, from 2018 to 2021 (i.e., severe acute respiratory infection). The nasopharyngeal swabs were collected in universal transport medium (Copan, Brescia, Italy) and stored at −80 °C until use. To confirm amplification specificity, we used pharyngeal, nasopharyngeal, and nasal swabs from Discovery Life Sciences (Los Osos, CA, USA) that were obtained for the Japanese national surveillance program. The study protocol was approved by the Ethics Committees of the National Institute of Infectious Diseases (Nos. 1001 and 1087) and Fukushima Medical University (No. 29006).

### 4.2. Real-Time RT-PCR for Detection of RSV 

RNA was extracted using the QIAamp Viral RNA Mini Kit (Qiagen, Hilden, Germany), QIAamp 96 Virus QIAcubeHT Kit (Qiagen), or Nucleospin 96 Virus Kit (Macherey-Nagel, Düren, Germany) in accordance with the manufacturers′ instructions. The US-CDC Pan-RSV assay [7] and duplex assay [8] were used in this study. The primers and probe for the Pan-RSV assay were as follows: forward (final concentration 500 nM), 5′-GGCAAATATGGAAACATACGTGAA-3′; reverse (250 nM), 5′-TCTTTTTCTAGGACATTGTAYTGAACAG-3′; probe (50 nM), 5′-FAM-CTGTGTATGTGGAGCCTTCGTGAAGCT-BHQ-3′ [7]. The primers and probe for the duplex assay were as follows: forward (200 nM), 5′-ATGGCTCTTAGCAAAGTCAAGT-3′; reverse (200 nM), 5′-TGCACATCATAATTRGGAGTRTCA-3′; probe for A (50 nM), 5′-FAM-ACACTCAACAAAGAT(BHQ1)CAACTTCTRTCATCCAGCA-phosphate-3′; probe for B (50 nM) 5′-Texas-Red-ACATTAAATAAGGAT(BHQ2)CAGCTGCTGTCATCCAGCA-phosphate-3′ (internal T modified with a BHQ quencher) [8]. Real-time RT-PCR was performed using AgPath-ID One-Step RT-PCR Reagent (AgPath-ID, Thermo Fisher Scientific) on a LightCycler 96 or LightCycler480 II instrument (Roche, Basel, Switzerland), under the following conditions: 50 °C for 600 s; 95 °C for 600 s; and 40 cycles of 95 °C for 15 s, 56 °C for 30 s, and 72 °C for 15 s. To avoid false-positives in the Pan-RSV assay, Cp values < 35 were considered positive [10]. Under these amplification conditions, non-specific amplifications occurred even in the negative controls, but Cp values < 35 could exclude all false-positives. The Pan-RSV assay was also performed using the reported conditions [8]. To validate the duplex assay, control RNA templates were amplified by RT-PCR (A, long; B, CH-18537) using the following primers: forward for A, 5′-TAATACGACTCACTATAGGGAAACCAAGATTCAAACAATC-3′; reverse for A, 5′-CAAGCTATTTAGGTGACACTATAGGCTAATGTTAACACTTCAAA-3′; forward for B, 5′-TAATACGACTCACTATAGGGGACCTCAACCCGTAACTTCC-3′; reverse for B, 5′-CAAGCTATTTAGGTGACACTATAGTTCATTTCCTTTCCATTTATA-3′. To validate primer/probe mismatches, PCR amplicons from mismatched specimens were used. RNA was synthesized from purified PCR amplicons using Riboprobe Systems (SP6, Promega, Madison, WI, USA). The copy numbers of the control RNAs were calculated based on the molecular weight and the absorbance at 260 nm of RNA that had been serially diluted (≥3 steps) and/or fluorescence using the QuantiFluor RNA system and Quantus Fluorometer (Promega) (*n* = 3). The copy-number-adjusted control RNA was diluted with PCR-grade water containing RNase inhibitor (1 U/μL) and yeast RNA (10 ng/mL) for storage at −80 °C. In this study, analytical sensitivity was defined as the mean (*n* = 4) of the 50% detectable copy number (per reaction) calculated by the Reed–Müench method.

### 4.3. Evaluation of Primer/Probe Mismatches with the Pan-RSV and Duplex Assays 

The nearly complete genomic sequences of RSV deposited in the GenBank database were collected via the NCBI virus (https://www.ncbi.nlm.nih.gov/labs/virus/vssi/#/, accessed on 28 April 2022) using its taxid (11,250). Each sequence was aligned using the multiple alignment program for amino acid or nucleotide sequences (MAFFT version 7, https://mafft.cbrc.jp/alignment/server/add_fragments.html?frommanual, accessed on 28 April 2022) [19]. Mismatches in targeted region sequences were analyzed using SEQUENCHER software (Gene Codes, Ann Arbor, MI, USA) with these alignments. Sequences containing “N” in the targeted region were excluded from the analysis.

### 4.4. Sequencing Analysis 

For Sanger sequencing of the Pan-RSV assay-targeted region, reverse transcription was performed using SMART M-MLV Reverse Transcriptase (TaKaRa Bio, Shiga, Japan) and random primers, in accordance with the manufacturer′s instructions. PCR amplification was performed using Quick Taq HS DyeMix (Toyobo, Osaka, Japan) with the primers used to amplify the control templates. Cycle sequencing was performed with BigDye Terminator v. 3.1 (Thermo Fisher Scientific) and a T7 primer on the 3500xl Genetic Analyzer (Thermo Fisher Scientific). Phylogenetic analysis was performed using MEGA-X software (ver. 10.1.8) [20] and sequences from GenBank (accession Nos. LC474522–LC474560) [11]. For NGS analysis, clinical isolates of RSV were obtained from HBTEC-ALI cultures prepared as described previously [12]. Direct NGS analysis of clinical specimens is ethically prohibited to avoid the disclosure of personal information. Therefore, a cell wash of RSV-infected HBTEC-ALI cultures was used to mimic the clinical swabs. RSV-positive clinical specimens were inoculated onto HBTEC-ALI cultures with various antibiotics (penicillin-streptomycin, gentamicin, and fangizone). After overnight inoculation, the cells were washed three times with the culture medium, and after 7 days of incubation cell washes were collected. Virus replication was confirmed using real-time RT-PCR assays. RNA was extracted from four RSV-replicated samples using ISOGEN-LS reagent (Nippongene, Tokyo, Japan). Libraries were prepared using the NEBNext Ultra II RNA Library Preparation Kit for Illumina (New England Biolabs, Ipswich, MA, USA), in accordance with the manufacturer′s instructions. The indexed libraries were analyzed for 2 × 150 cycles on a DNBSEQ-G400 instrument at GENEWIZ (South Plainfield, NJ, USA). Reads were trimmed and de-novo assembled using CLC Genomics Workbench v. 21.0.4 with default settings. The gene annotations were analyzed by VAPiD v1.6.6 [21]. The coverage of the assembled sequences was checked by mapping for the targeted region sequences of the Pan-RSV and duplex assays and mRNA encoding the N and M proteins derived from the RSV isolates in 2014 (LC474558 and LC474559).

### 4.5. Statistical Analysis 

*z*-, chi-squared, and *t*-tests were performed using SigmaPlot v. 14.5 (Systat Software Inc., San Jose, CA, USA). *p*-values < 0.05 were considered indicative of statistical significance.

## Figures and Tables

**Table 1:** The number of mismatches in the primers/probes of the Pan-RSV and duplex assays using registered sequences.

		Year Registered	All	2010–2015	2016–2022
		Total number	2935	1069	1185
Pan-RSV	Forward	Number of mismatched sequences	60 (2.0%)	4 (0.37%)	6 (0.5%)
		(Within 10 nucleotides from the 3′-end)	57 (1.9%)	3 (0.28%)	6 (0.5%)
	Reverse	Number of mismatched sequences	1457 (49.7%)	456 (42.7%)	703 (59.3%)
		(Within 10 nucleotides from the 3′-end)	275 (9.4%)	10 (0.9%)	257 (21.7%)
	Probe	Number of mismatched sequences	1579 (53.8%)	623 (58.3%)	544 (45.9%)
Duplex	Forward	Number of mismatched sequences	N.D. *	N.D.	25 (2.1%)
		(Within 10 nucleotides from the 3′-end)	N.D.	N.D.	18 (1.5%)
	Reverse	Number of mismatched sequences	N.D.	N.D.	34 (2.9%)
		(Within 10 nucleotides from the 3′-end)	N.D.	N.D.	3 (0.3%)

* N.D., not determined.

**Table 2:** Mismatches in the primers/probe of the Pan-RSV assay for subgroup A.

Sequence Pattern	Forward	Probe	Reverse	Number/Total Reads
	(5′ → 3′)	(3′ ← 5′)	(3′ ← 5′)	
	GGCAAATATGGAAACATACGTGAA	AGCTTCACGAAGGCTCCACATACACAG	CTGTTCARTACAATGTCCTAGAAAAAGA	
1	- *	A**A**CTTCACGA**G**GGCTCCACATACACAG	-	9/10
2	-	A**A**CTTCACGA**G**GGCTCCACATACACAG	CTGTTCAATACAATGTCCT**G**GAAAAAGA	1/10

* no mismatch.

**Table 3:** Mismatches in the primers/probe of the Pan-RSV assay for subgroup B.

Sequence Pattern	Forward	Probe	Reverse	Number/Total Reads
	(5′ → 3′)	(3′ ← 5′)	(3′ ← 5′)	
	GGCAAATATGGAAACATACGTGAA	AGCTTCACGAAGGCTCCACATACACAG	CTGTTCARTACAATGTCCTAGAAAAAGA	
1	- *	-	CTGTTCAGTACAATGT**T**CTAGAAAAAGA	20/78
2	GGCAAATATGGAAACA**C**ACGTGAA	-	CTGTTCAGTACAATGT**T**CTAGAAAAAGA	1/78
3	-	-	CTGTTCAGTACAATGT**TA**TAGAAAAAGA	2/78
4	-	-	CTGT**C**CAGTACAATGT**T**CTAGAAAAAGA	46/78
5	-	-	CTGTTCAGTACAATGT**T**CTAGAAAAGGA	2/78
6	-	-	CTGT**C**CAGTACAATGT**T**CT**G**GA**G**AAAGA	1/78
7	-	AGCTTCACGAAG**A**CTCCACATACACAG	CTGTTCAGTACAATGT**T**CTAGAAAAAGA	1/78
8	-	AGCTTCACGAAG**A**CTCCACA**C**A**A**ACAG	CTGTTCAGTACAATGT**T**CTAGAAAAAGA	3/78
9	-	AGCTTCACGAAGGCTCCACA**C**A**A**ACAG	CTGTTCAGTACAATGT**T**CTAGAAAAAGA	2/78

* no mismatch.

**Table 4:** Analytical sensitivities of the Pan-RSV assay for the mismatched sequences in subgroup B.

Sequence Name	Mismatch(es)	Analytical Sensitivity (Copies)	*p*-Value (vs. 1391/13)
RSV/B/Sendai/1391/13	Forward primer (CTGTTCAGTACAATGT**T**CTAGAAAAAGA)	1.1 ± 0.6	
RSV/B/Sendai/125/15	Probe (AGCTTCACGAAG**A**CTCCACATACACAG)	1.7 ± 1.6	0.374
RSV/B/Sendai/1008/15	Probe (AGCTTCACGAAG**A**CTCCACA**C**A**A**ACAG)	0.8 ± 0.0	0.500
RSV/B/Sendai/1060/16	Probe (AGCTTCACGAAGGCTCCACA**C**A**A**ACAG)	2.0 ± 2.6	0.296
RSV/B/Sendai/442/17	Forward primer (CTGT**C**CAGTACAATGT**T**CTAGAAAAAGA)	1.4 ± 1.3	0.515

**Table 5:** Coverage of the targeted region of the Pan-RSV and duplex assays for RSV isolates.

		Average Coverage for the Targeted Region				
Isolate Name	Subgroup	Pan-RSV	Duplex Assay	mRNA for M	mRNA for N	Run ID *
Fukushima_OR343_2021	A	37	515	63914	99287	DRR353553
Fukushima_OR371_2021	A	94	629	93792	110272	DRR353554
Fukushima_OR358_2021	B	52	1391	95320	87481	DRR353555
Fukushima_OR379_2021	B	82	2841	186829	236367	DRR353556

* Bioproject: PRJDB13228.

## Data Availability

This study used almost-complete genomic RNA sequences of RSV isolates from Sendai, Japan, from 2010 to 2017 [11], as determined by Agoti′s method [14], (GenBank accession nos. LC474522-LC474560). This study also used almost-complete sequences of RSV strains isolated by HBTEC-ALI culture (LC69933–LC69936). The raw read data are available in BioProject (PRJDB13228). The run IDs are RSV_A_Fukushima_OR343_2021, DRR353553; RSV_A_Fukushima_OR371_2021, DRR353554; RSV_B_Fukushima_OR358_2021, DRR353555; and RSV_B_Fukushima_OR379_2021, DRR353556.
